# Emerging patterns and trends in global cancer burden attributable to metabolic factors, based on the Global Burden of Disease Study 2019

**DOI:** 10.3389/fonc.2023.1032749

**Published:** 2023-01-19

**Authors:** Yan Zhang, Yuwei Ding, Ning Zhu, Mi Mi, Yier Lu, Jia Zheng, Shanshan Weng, Ying Yuan

**Affiliations:** ^1^ Department of Medical Oncology (Key Laboratory of Cancer Prevention and Intervention, China National Ministry of Education, Key Laboratory of Molecular Biology in Medical Sciences, Zhejiang Province, China), The Second Affiliated Hospital, Zhejiang University School of Medicine, Hangzhou, Zhejiang, China; ^2^ Zhejiang Provincial Clinical Research Center for CANCER, Hangzhou, Zhejiang, China; ^3^ Department of Medical Geriatrics, School of Medicine, The Second Affiliated Hospital of Zhejiang University, Hangzhou, Zhejiang, China; ^4^ Cancer Center, Zhejiang University, Hangzhou, Zhejiang, China

**Keywords:** global burden of disease study (GBD), cancer, mortality, age standardized rate (ASR), average annual percentage change (AAPC)

## Abstract

**Background:**

The exponential growth of the cancer burden attributable to metabolic factors deserves global attention. We investigated the trends of cancer mortality attributable to metabolic factors in 204 countries and regions between 1990 and 2019.

**Methods:**

We extracted data from the Global Burden of Disease Study (GBD) 2019 and assessed the mortality, age-standardized death rate (ASDR), and population attributable fractions (PAFs) of cancers attributable to metabolic factors. Average annual percentage changes (AAPCs) were calculated to assess the changes in the ASDR. The cancer mortality burden was evaluated according to geographic location, SDI quintiles, age, sex, and changes over time.

**Results:**

Cancer attributable to metabolic factors contributed 865,440 (95% UI, 447,970-140,590) deaths in 2019, a 167.45% increase over 1990. In the past 30 years, the increase in the number of deaths and ASDR in lower SDI regions have been significantly higher than in higher SDI regions (from high to low SDIs: the changes in death numbers were 108.72%, 135.7%, 288.26%, 375.34%, and 288.26%, and the AAPCs were 0.42%, 0.58%, 1.51%, 2.36%, and 1.96%). Equatorial Guinea (AAPC= 5.71%), Cabo Verde (AAPC=4.54%), and Lesotho (AAPC=4.42%) had the largest increase in ASDR. Large differences were observed in the ASDRs by sex across different SDIs, and the male-to-female ratios of ASDR were 1.42, 1.50, 1.32, 0.93, and 0.86 in 2019. The core population of death in higher SDI regions is the age group of 70 years and above, and the lower SDI regions are concentrated in the age group of 50-69 years. The proportion of premature deaths in lower SDI regions is significantly higher than that in higher SDI regions (from high to low SDIs: 2%, 4%, 7%, 7%, and 9%). Gastrointestinal cancers were the core burden, accounting for 50.11% of cancer deaths attributable to metabolic factors, among which the top three cancers were tracheal, bronchus, and lung cancer, followed by colon and rectum cancer and breast cancer.

**Conclusions:**

The cancer mortality burden attributable to metabolic factors is shifting from higher SDI regions to lower SDI regions. Sex differences show regional heterogeneity, with men having a significantly higher burden than women in higher SDI regions but the opposite is observed in lower SDI regions. Lower SDI regions have a heavier premature death burden. Gastrointestinal cancers are the core of the burden of cancer attributable to metabolic factors.

## Introduction

Cancer is a major public health challenge, with 19.29 million new cases and 9.96 million deaths in 2020 (1). Related risk factors play a key role in the occurrence and development of cancer, such as hypertension, tobacco use, and air pollution. The cancer mortality burden attributable to these factors is preventable and controllable (2). In 2014, cancer deaths among Chinese adults aged 20 and above accounted for 45.2% of all deaths (3). The prevention and control of cancer mortality is the top priority in reducing the global cancer burden. In recent years, the cancer burden attributable to tobacco (4) and alcohol use (5) has decreased significantly under effective policies to control tobacco and alcohol use. Although there is heterogeneity in the cancer burden attributable to dietary risks (heterogeneity of regions and cancer types), the overall cancer burden attributable to dietary risks has decreased (6–9). In general, due to the increase in body weight of the global population and the poor control of metabolic-related diseases (such as diabetes), the related cancer mortality burden has shown an upward trend to varying degrees (2).

Motivated by the increasing occurrence of metabolic diseases and the related mortality burden of cancer, this study extracted data from the Global Burden of Disease (GBD) 2019 study and explored the distribution and time trends of the cancer burden attributable to metabolic factors in different regions, countries, sexes, and age groups to formulate appropriate strategies for prevention and control.

## Materials and methods

### Data sources

The data used in this study were collected by the Global Burden of Disease Study 2019 using the Global Heath Data Exchange Tool (https://ghdx.healthdata.org/gbd-results-tool). Deaths, age-standardized death rate (ASDR), summary exposure values (SEVs), population attributable fractions (PAFs) and their 95% uncertainty intervals (UIs) from 1990 to 2019 among 5 sociodemographic index (SDI) quintiles, 21 GBD regions, and 204 countries and territories were extracted. Temporal trends in death rates were assessed by the average annual percentage change (AAPC), which was calculated based on the Joinpoint regression model.

SDI is an indicator to measure the lagging distribution of a country’s overall fertility rate, education level and per capita income, ranging from 0 to 1. According to their SDI values in 2019, countries and regions were divided into five categories (high>0.81, medium high 0.70-0.81, medium high 0.61-0.69, medium low 0.46-0.60 and low<0.46).

Cancers are classified according to the International Classification of Diseases (ICD). All cancer death rates were obtained from a single cancer registry or a summary database of cancer registries, including the cancer incidence (CI5), surveillance, epidemiology and final results (SEER) on five continents, and the Nordic Cancer Registry (NORDCAN). The classification of cancers attributable to metabolic factors retrieved from the GBD data includes 15 kinds: breast cancer, colon and rectum cancer, liver cancer, ovarian cancer, pancreatic cancer, esophageal cancer, gallbladder and malignant tract cancer, kidney cancer, leukemia, multiple myeloma, non-Hodgkin lymphoma, thyroid cancer, uterine cancer, bladder cancer, and tracheal-bronchus and lung cancer (10, 11).

### Attributable risk factors

The first-level risk factors in GBD are divided into behavioral, metabolic, and environmental/occupational risks. Attributable risks of cancer were estimated to be 11 risk factors: air pollution, occupational risks, and other environmental risks, which are environmental factors; tobacco, dietary risks, alcohol use, unsafe sex, drug use, and low physical activity, which are behavioral factors; and high fasting plasma glucose and high body mass index, which are metabolic factors (2).

The summary exposure values (SEVs) of the metabolic factors (including high fasting plasma glucose and high body mass index), the population attributable fractions (PAFs) of various cancers, and the cancer burden and trend attributable to metabolic factors were analyzed.

### Statistical analysis

The age-standardized rate (ASR) is expressed as the number of people per 100 000 population. Regression analysis was performed to calculate the average annual percentage change (AAPC) and 95% confidence interval (CI) for the average standardized death rate (ASDR) using Joinpoint 4.2.0.1 software. The joinpoint regression model is used to evaluate the time trend in a structured way and test that the trend between joint points is statistically significant (12, 13). When the slope of the trend (AAPC) is significantly different from zero, the terms “decrease” or “increase” are used. R language software was used to analyze the correlation between ASDR and SDI in 21 GBD regions and to plot the changes in the number of deaths, ASDR and AAPC map distribution in 204 countries and territories. P values less than 0.05 were considered statistically significant.

## Results

### In contrast to other risk factors, the cancer mortality burden attributable to metabolic factors shows a significant upward trend worldwide

**Figure 1:**
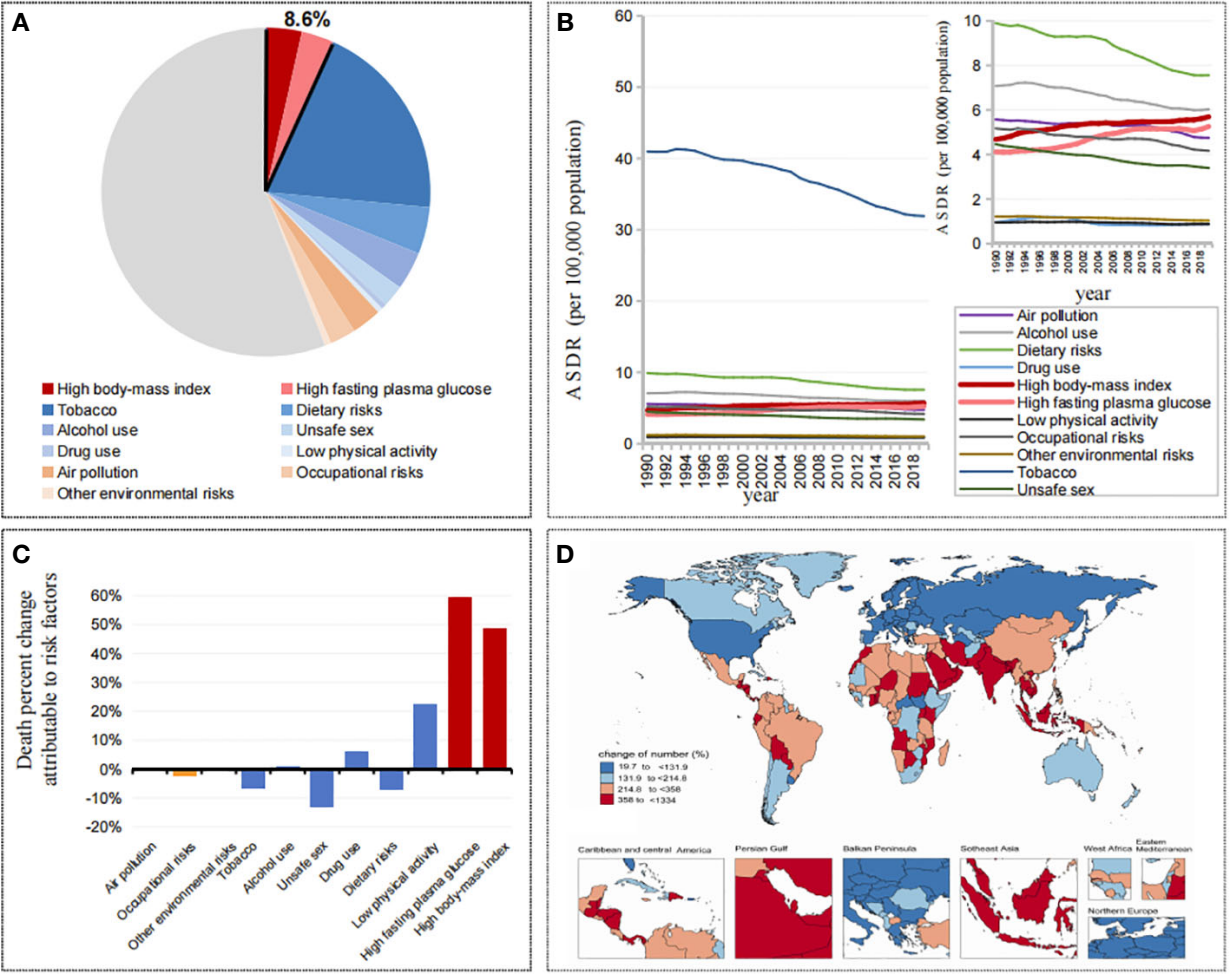
**(A)** Contributions of attributable death cases by different risk factors in 2019. **(B)** The trend of ASDRs in cancers attributable to different risk factors from 1990 to 2019. **(C)** Changes of death percent in cancers attributable to different risk factors from 1990 to 2019. **(D)** Changes of death cases in cancers attributable to metabolic factors by countries and territories from 1990 to 2019.

Globally, attributable cancer mortality accounted for 44.16% of all cancer mortality, of which metabolic factors accounted for 8.59% (high body-mass index accounted for 4.59%, high fasting plasma glucose accounted for 4.16%), behavior factors accounted for 36.70% (tobacco accounted for 25.80%, dietary risks accounted for 6.01%, alcohol use accounted for 4.91%, low physical activity accounted for 0.70%), and environmental risks accounted for 7.31% (**Figure 1A**). In the past 30 years, among these attributable cancer mortality burdens, the related risks represented by tobacco and alcohol use showed a significant downward trend, while metabolic factors, including high body mass index and high fasting plasma glucose, were different from all of the other factors, showing a significant increasing trend (**Figure 1B**). Meanwhile, 5 SDIs and 21 GBD regions showed the same trend (**Figure S1**).

From 1990 to 2019, in the death percent change attributable to risk factors, metabolic factors increased significantly, among which high fasting plasma glucose was 59.60%, high body mass index was 48.55%, and low physical activity was 22.24%, while alcohol use, tobacco and dietary risks decreased by -0.83%, -6.84%, and -7.17%, respectively (**Figure 1C**). Among the 204 countries and territories, the vast majority of countries (113/204) showed a doubling trend in the number of cancer deaths attributable to metabolic factors, with the fastest growth in the United Arab Emirates (1334.02%), Djibouti (930.67%) and Qatar (913.30%), and with the smallest increase in Kyrgyzstan (42.18%), Kazakhstan (35.53%) and Ukraine (19.73%) (**Figure 1D**).

### The cancer mortality burden attributable to metabolic factors is shifting from the higher SDI regions to the lower SDI regions

**Figure 2:**
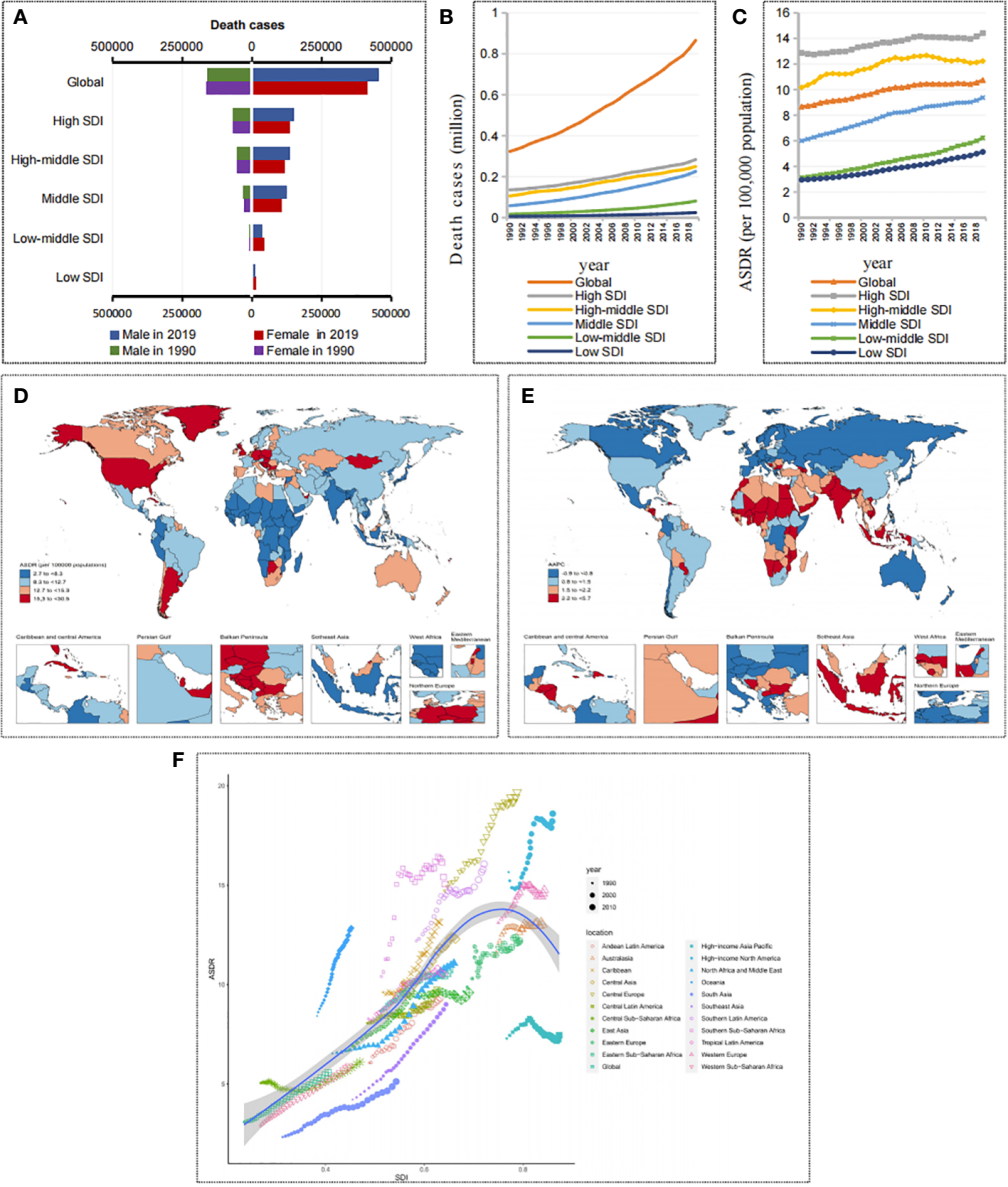
Death burden in cancers attributable to metabolic factors. **(A)** Death cases in cancers attributable to metabolic factors by SDIs and sexes in 1990 and 2019. **(B)** The trend of death cases in cancers attributable to metabolic factors by SDIs from 1990 to 2019. **(C)** The trend of ASDRs in cancers attributable to metabolic factors by SDIs from 1990 to 2019. **(D)** ASDRs of cancers attributable to metabolic factors by countries and territories in 2019. **(E)** AAPC of ASDRs in cancers attributable to metabolic factors by countries and territories from 1990 to 2019. **(F)** ASDRs of cancers attributable to metabolic factors by GBD regions from 1990 to 2019.

Over the past 30 years, the number of cancer deaths attributable to metabolic factors has doubled worldwide, from 323,590 (95% UI, 151,880-545,070) to 865,440 (95% UI, 447,970-1,405,900) (**Figures 2A, B** and **Table 1**). The ASDR increased from 8.67 (95% UI, 4.07-14.66) per 100,000 people in 1990 to 10.74 (95% UI, 5.53-17.5) per 100,000 people in 2019 (**Figure 2C** and **Table 1**), and the AAPC was 0.74% (95% UI, 0.71%-0.76%) (**Table 1**). Notably, the prevalence patterns of cancer mortality burden attributable to metabolic factors changed in differently in different SDI regions.

**Table 1:** The death cases, ASDR of cancers attributable to metabolic factors in 1990 and 2019, and their variations from 1990 to 2019.

	Deaths			ASDR		
Location	1990 (95% UI) (No. ×10^3^)	2019 (95% UI) (No. ×10^3^)	Changes of number (%)	1990 (95% UI) ^a^	2019 (95% UI) ^a^	AAPC (95%CI)
Global	323.59 (151.88~545.07)	865.44 (447.97~1405.9)	167.45	8.67(4.07~14.66)	10.74 (5.53~17.5)	0.74 (0.71~0.76)
Socio-demographic index						
High SDI	135.89 (66.91~225.4)	283.62 (148.59~454.93)	108.72	12.87 (6.36~21.28)	14.42 (7.7~22.97)	0.42 (0.41~0.44)
High-middle SDI	105.97 (52.56~173.12)	249.77 (132.68~401.11)	135.7	10.17 (5.02~16.66)	12.24(6.48~19.67)	0.58 (0.53~0.64)
Middle SDI	58.1 (22.98~108.35)	225.59 (111.5~373.6)	288.26	6.01 (2.35~11.17)	9.38 (4.57~15.62)	1.51 (1.47~1.55)
Low-middle SDI	17.1 (6.35~31.6)	81.27 (38.35~136.63)	375.34	3.13 (1.16~5.82)	6.23 (2.89~10.55)	2.36 (2.34~2.37)
Low SDI	6.35 (2.44~11.66)	24.67 (11.59~41.16)	288.32	2.96 (1.11~5.48)	5.14 (2.35~8.71)	1.96 (1.95~1.98)
GBD Regions						
High-income Asia Pacific	14.31 (5.24~26.73)	36.52 (13.8~67.16)	155.19	7.28 (2.64~13.62)	7.45 (2.88~13.53)	-0.28 (-0.33~-0.23)
Central Asia	4.48 (2.48~6.97)	8.7 (5.02~13.18)	94.23	9.61 (5.32~14.98)	12.3 (7.09~18.71)	1.01 (0.95~1.08)
East Asia	56.46 (17.71~112.21)	196.02 (82.03~349.24)	247.19	6.7 (2.11~13.35)	9.6 (3.97~17.16)	1.2 (1.12~1.28)
Southeast Asia	10.11 (3.76~18.65)	52.2 (25.69~88.22)	416.28	4.22 (1.54~7.84)	9 (4.3~15.47)	2.57 (2.55~2.59)
South Asia	11.64 (4.17~21.82)	68.69 (31.27~115.45)	489.95	2.33 (0.82~4.41)	5.12 (2.3~8.66)	2.61 (2.57~2.65)
Australasia	2.67 (1.42~4.2)	6.69 (3.86~10.3)	150.54	11.43 (6.07~17.98)	13.02 (7.52~19.8)	0.28 (0.25~0.32)
Oceania	0.24 (0.11~0.41)	0.89 (0.41~1.54)	265.39	8.42 (3.77~14.41)	12.84 (6~22.48)	1.4 (1.38~1.43)
North Africa and Middle East	10.64 (5.59~16.96)	44.85 (25.65~70.51)	321.48	6.53 (3.4~10.45)	11.08 (6.21~17.48)	2.19 (2.12~2.26)
Central Sub-Saharan Africa	1.03 (0.44~1.94)	2.9 (1.28~5.31)	181.56	5.05 (2.09~9.7)	6.11 (2.64~11.24)	0.4 (0.3~0.49)
Eastern Sub-Saharan Africa	2.13 (0.82~3.93)	8.38 (4.19~13.79)	293.27	3.09 (1.16~5.68)	5.57 (2.72~9.24)	2.2 (2.17~2.23)
Southern Sub-Saharan Africa	2.69 (1.47~4.26)	7.87 (4.67~11.68)	192.23	10.41 (5.63~16.57)	15.05 (8.92~22.37)	1.21 (1.08~1.34)
Western Sub-Saharan Africa	2.3 (0.99~4.09)	9.81 (5.03~15.97)	326.61	2.89 (1.2~5.18)	5.88 (3~9.65)	2.54 (2.53~2.55)
Central Europe	21.64 (12.02~33.65)	43.17 (24.26~68.03)	99.52	14.72 (8.18~22.92)	19.69 (11.04~30.99)	1.09 (1.05~1.12)
Eastern Europe	27.43 (15.9~41.08)	42.65 (26.01~63.12)	55.48	9.65 (5.6~14.44)	12.2 (7.44~18.04)	0.57 (0.51~0.63)
Western Europe	77.62 (38.6~127.13)	142.88 (72.18~230.69)	84.08	13.11 (6.51~21.43)	14.76 (7.53~23.73)	0.31 (0.27~0.35)
High-income North America	55.65 (27.82~91.85)	120.05 (66.13~187.74)	115.72	15.66 (7.86~25.75)	18.6 (10.33~28.92)	0.91 (0.86~0.95)
Caribbean	2.44 (1.22~3.98)	6.81 (3.64~11.08)	179.57	9.61 (4.8~15.76)	13.13 (7.01~21.35)	1.26 (1.22~1.31)
Andean Latin America	1.2 (0.64~1.88)	5.11 (2.9~8.09)	325.91	6.06 (3.19~9.6)	9.31 (5.27~14.77)	1.59 (1.56~1.62)
Central Latin America	6.28 (3.17~10.23)	22.51 (12~36.5)	258.58	8.06 (3.99~13.21)	9.72 (5.18~15.79)	0.51 (0.48~0.54)
Tropical Latin America	7.08 (3.56~11.71)	25.14 (14.45~38.28)	255.19	8.28 (4.09~13.7)	10.54 (6.02~16.06)	1 (0.96~1.03)
Southern Latin America	5.54 (2.72~9.04)	13.59 (7.33~21.33)	145.15	12.2 (5.98~19.91)	16.07 (8.69~25.16)	0.74 (0.69~0.79)

ASDR, age-standardized death rate; AAPC, average annual percentage change; aPer 100,000 population; GBD, Global Disease Burden; SDI, Socio-demographic Index; UI, uncertainty interval.

In 2019, the number of cancer deaths attributable to metabolic factors from high to low SDI regions was 0.28, 0.25, 0.23, 0.08, and 0.02 million, and the ASDRs were 14.42, 12.23, 9.38, 6.23, and 5.14 per 100,000 people, respectively (**Figures 2B, C**). It is obvious that higher SDI regions have a higher mortality burden than lower SDI regions. However, in the past 30 years, there has been a more significant growth trend in lower SDI regions. The change in death numbers from high to low SDI regions was 108.72%, 135.7%, 288.26%, 375.34%, and 288.32%, and the AAPCs of the ASDR were 0.42%, 0.58%, 1.51%, 2.36%, and 1.96%, respectively (**Figure 2C** and **Table 1**). Based on the significant growth trend in lower SDI regions, the gap in mortality burden is narrowing compared with higher SDI regions. In other words, the cancer mortality burden attributable to metabolic factors is gradually shifting from higher SDI regions to lower SDI regions.

Among the 21 GBD regions, the trends were consistent with the 5 SDI regions, with mortality rates trending upward in most GBD regions over the past 30 years. However, the high-income Asia Pacific showed a downward trend (AAPC of ASDR = -0.28%). In contrast, lower SDI regions such as South Asia, Southeast Asia, Western Sub-Saharan Africa, North Africa and the Middle East showed a clear upward trend in mortality, with South Asia showing the most obvious upward trend (changes in death numbers=489.95%, AAPC of ASDR = 2.61%) (**Table 1** and **Figure 2F**).

Among the 204 countries and territories, the countries with the top three ASDRs in 2019 were Qatar, Mongolia and American Samoa, and the countries with the bottom three ASDRs were Bangladesh, Niger and Somalia. In the past 30 years, 73 countries had an increase of more than 300% in cancer deaths attributable to metabolic factors, 71 countries had AAPCs of ASDR greater than 2%, and the vast majority of these countries were concentrated in lower SDI regions (**Figures 2D, E**).

### Significant regional heterogeneity in sex differences was observed in the cancer mortality burden attributable to metabolic factors

**Figure 3:**
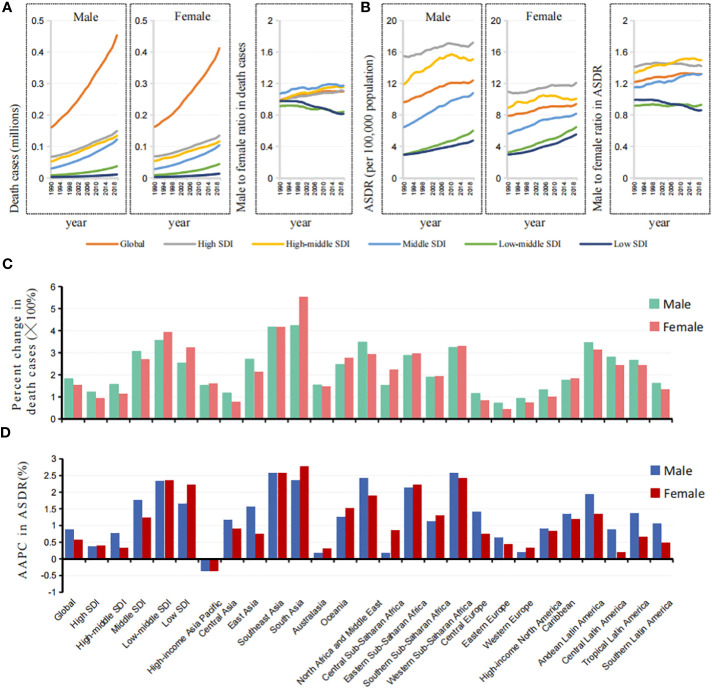
Sex differences and trends of ASDR in cancers attributable to metabolic factors by different SDIs and GBD regions. **(A)** The death cases in cancers attributable to metabolic factors in males and females, and male to female ratios by SDIs from 1990 to 2019. **(B)** The ASDRs in cancers attributable to metabolic factors in males and females, and male to female ratios by SDIs from 1990 to 2019. **(C)** The changes of death cases in cancers attributable to metabolic factors in males and females by SDIs and GBD regions from 1990 to 2019. **(D)** The AAPCs of ASDR in cancers attributable to metabolic factors in males and females by SDIs and GBD regions from 1990 to 2019.

In 2019, there were 0.453 million cancer deaths attributable to metabolic factors in men and 0.412 million in women, which were 2.82 times and 2.54 times those in 1990, respectively (**Figure 3A**). From 1990 to 2019, the ASDR increased from 9.63 to 12.39 per 100,000 people (AAPC=0.878%) in men and 7.90 to 9.38 per 100,000 people (AAPC=0.587%) in women (**Figures 3B, D**). It is worth noting that there is regional heterogeneity in sex differences.

In higher SDI regions, such as high, high-middle, and middle SDI regions, the male-to-female ratio of the death number and ASDR were greater than 1, and the values showed a clear upward trend in the past 30 years, which was completely opposite to the findings in the low-middle and low SDI regions (**Figures 3A, B**).

Among the 21 GBD regions, the percent changes in death cases and ASDR attributable to metabolic factors in women were significantly higher than those in men in South Asia, Oceania, and most areas of Africa, while the regions located in North Africa and the Middle East were the opposite (**Figures 3C, D**, **S2**).

It is clear that the metabolic cancer burden in men was greater than that in women in higher SDI regions, the exact opposite of the findings in lower SDI regions. Moreover, the sex gap between regions is widening.

### The cancer mortality burden attributable to metabolic factors tends to affect younger people in lower SDI regions

To analyze the trends in death across age groups, starting at age 20, we divided every five years into an age group for a total of 14 age groups.

**Figure 4:**
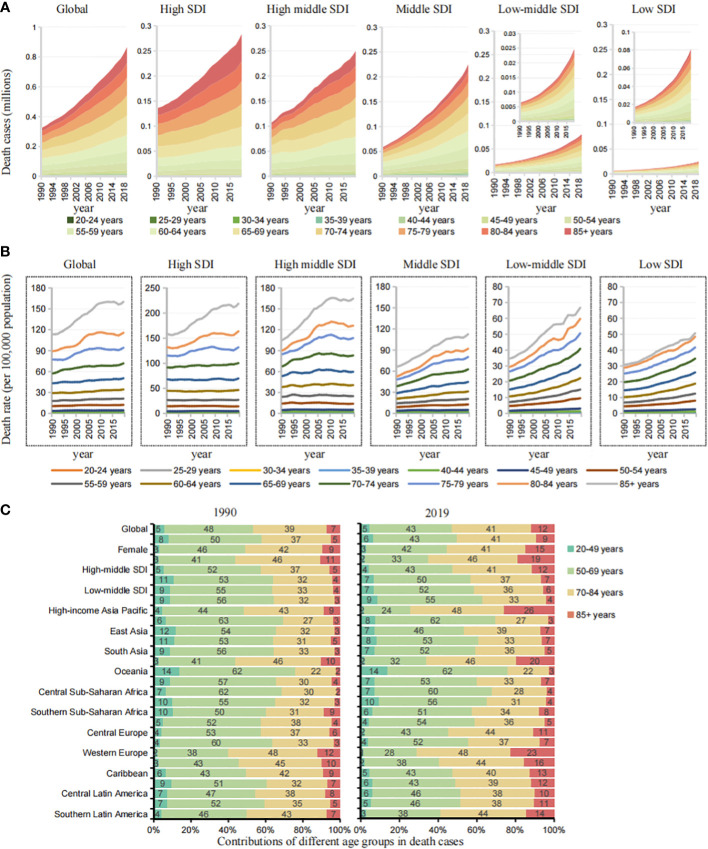
Age group differences and trends of cases in cancers by different SDIs and GBD regions. **(A)** The death cases trend of age groups in cancers attributable to metabolic factors by SDIs from 1990 to 2019. **(B)** The death rate trend of age groups in cancers attributable to metabolic factors by SDIs from 1990 to 2019. **(C)** Contributions of four age groups to cancer deaths attributable to metabolic factors by SDIs and GBD regions in 1990 and 2019.

In higher SDI regions (high, high-middle SDI), the mortality burden was mainly concentrated in the age group of 70 years and above. In lower SDI regions, deaths tended to be at younger ages. The proportion of premature deaths under the age of 50 was significantly higher than that in high SDI regions (**Figure 4A**). It is worth mentioning that in lower SDI regions, doubling trends in all age groups were observed, which was more obvious in the age group of 50 years and above (**Figure 4B**).

To further analyze regional differences in age, we simplified the age groups into four groups: 20-49, 50-69, 70-84, and ≥85 years (**Figures 4C**, **S3**). In the lower SDI regions (middle, middle-low, low SDI), the age group of 20-49 years accounted for 7%, 7%, and 9% of deaths in 2019, an increase of 150.39%, 264.18%, and 292.23% over 1990. In the higher SDI regions (high, high-middle SDI), the age group of 20-49 years accounted for 2% and 4%, an increase of 54.9% and 88.1% over 1990, and the age group of 85 years and above accounted for 19% and 12%, significantly higher than that in lower SDI regions. The main mortality burden was concentrated in the 70-84 years age group in higher SDI regions and in the 50–69 years age group in the lower SDI regions. Among the 21 GBD regions, Oceania, Eastern sub-Saharan Africa and South Asia had low SDI values, and the age group of 20-49 years accounted for 14%, 10%, and 7% of the deaths in 2019, respectively, an increase of 274.38%, 278.06% and 542.77% over 1990.

In lower SDI regions, the cancer mortality burden attributable to metabolic factors tends to affect younger people, and the burden of premature death is more obvious.

### Gastrointestinal cancers are the major cancer mortality burden attributable to metabolic factors

Significant changes in the standardized exposure values (SEVs) of the high body mass index and high fasting plasma glucose occurred in the past 30 years, which are highly associated with the prevalent changes in the cancer mortality burden attributable to metabolic factors during these years (**Supplementary File 2**). To better explore the burden attributable to specific metabolic factors, we performed a trend analysis of two risk factors (a high body mass index and a high fasting plasma glucose) for different cancer types, 5 SDI regions, and 21 GBD regions.

**Figure 5:**
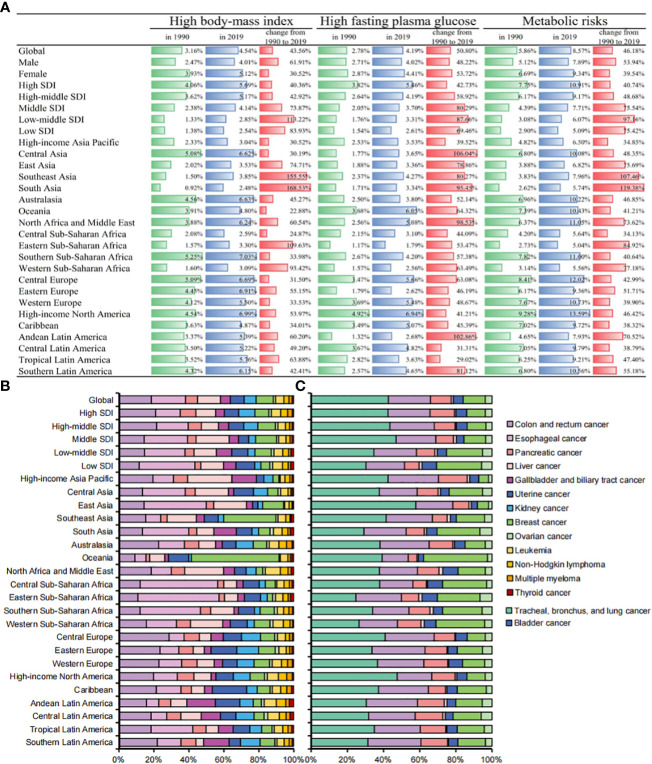
**(A)** Percentages of ASDRs in cancers attributable to metabolic factors by SDIs and GBD regions in 1990, 2019, and changes from 1990 to 2019. **(B)** Contributions of different cancers to total cancer deaths attributable to high-body mass index by SDIs and GBD regions in 2019. **(C)** Contributions of different cancers to total cancer deaths attributable to high fasting plasma glucose by SDIs and GBD regions in 2019.

Significant heterogeneity was observed in the cancer mortality burden attributable to a high body-mass index and a high fasting plasma glucose (**Figure 5A**). In 2019, the proportion of ASDR (from high to low SDI) attributable to a high body mass index was 5.69%, 5.17%, 4.14%, 2.85% and 2.54%, respectively, 40.36%, 42.92%, 73.87%, 113.22% and 83.93% higher than that in 1990, while the proportion of ASDR attributable to a high fasting plasma glucose was 5.46%, 4.19%, 3.70%, 3.31% and 2.61%, respectively, 42.73%, 58.92%, 80.29%, 87.66%, and 69.46% higher than that in 1990. Among the 21 GBD regions, lower SDI regions such as South Asia and Southeast Asia had the largest increases in ASDR attributable to a high body mass index (168.53% and 155.55%, respectively), while Central Asia and Andean Latin America had the largest increases in ASDR attributable to a high fasting plasma glucose (106.04% and 102.86%, respectively). Globally, there were more significant sex differences found for a high fasting plasma glucose than a high body mass index (**Figure S4**). Among the age proportions, the age group of 50-69 years was the core population of the cancer mortality burden attributable to a high body mass index, while the cancer mortality burden attributable to a high fasting plasma glucose was more concentrated in the age group of 70-84 years (**Figure S5**).

We further analyzed the cancer mortality burden attributable to metabolic factors in different cancers. Digestive system tumors (esophageal cancer, colon and rectum cancer, pancreatic cancer, liver cancer, cholangiocarcinoma) accounted for 50.11% (433,700 deaths) of all deaths. Digestive system cancers were the core burden of death attributable to metabolic factors. Esophageal cancer, colon and rectum cancer, and pancreatic cancer are the top three cancers attributable to a high body-mass index, with 89,900, 85,900, and 60,800 deaths in 2019, respectively. Tracheal, bronchus, and lung cancer, colon and rectum cancer, and breast cancer are the top three cancers attributable to a high fasting plasma glucose, with 179,000, 9,7600, and 51,100 deaths in 2019, respectively. Uterine cancer and kidney cancer had the highest proportion of deaths attributable to metabolic factors, accounting for 39.8% and 19.0% of all deaths from metabolic cancer, respectively (**Table 2**).

**Table 2:** Population attributable fraction of cancers by metabolic factors in 2019, and changes from 1990 to 2019.

	High body-mass index				High fasting plasma glucose				Metabolic factors			
	death cases in 2019	death cases change from 1990 to 2019 (%)	PAF in 2019(%	change of PAF from 1990 to 2019 (%)	death cases in 2019	death cases change from 1990 to 2019 (%)	PAF in 2019(%	change of PAF from 1990 to 2019 (%)	death cases in 2019	death cases change from 1990 to 2019 (%)	PAF in 2019(%)	change of PAF from 1990 to 2019 (%)
Tracheal, bronchus, and lung cancer	0	0	0	0	179048.8	166.2	8.8	38.9	179048.8	166.2	8.8	38.9
Colon and rectum cancer	85881.7	169.3	7.9	28.5	97582.1	186.3	9.0	36.6	175141.6	174.7	16.1	31.1
Esophageal cancer	89903.9	154.8	18.1	63.0	0	0	0	0	89903.9	154.8	18.1	63.0
Pancreatic cancer	31921.1	229.3	9.1	22.8	48358.5	270.1	14.5	38.0	77215.1	249.5	14.5	30.3
Liver cancer	60799.1	162.3	12.5	97.7	4729.5	137.1	1.0	79.0	65278.2	160.0	13.5	96.0
Gallbladder and biliary tract cancer	26126.8	105.6	15.2	13.1	0	0	0	0	26126.8	105.6	15.2	13.1
Uterine cancer	36485.8	112.3	39.8	29.9	0	0	0	0	36485.8	112.3	39.8	29.9
Kidney cancer	31704.9	183.2	19.0	22.7	0	0	0	0	31704.9	183.2	19.0	22.7
Bladder cancer	0	0	0	0	22823.3	167.2	10.0	42.1	22823.3	167.2	10.0	42.1
Breast cancer	45202.6	197.2	6.4	61.5	51059.9	160.4	7.3	41.7	91985.7	173.4	13.1	48.7
Ovarian cancer	6308.9	139.9	3.2	17.2	15735.9	184.2	7.9	39.4	21514.4	168.7	10.8	31.7
Leukemia	21733.8	132.5	6.5	82.2	0	0	0	0	21733.8	132.5	6.5	82.2
Non-Hodgkin lymphoma	13797.5	176.2	5.4	36.6	0	0	0	0	13797.5	176.2	5.4	36.6
Multiple myeloma	8019.0	188.4	7.1	31.8	0	0	0	0	8019.0	188.4	7.1	31.8
Thyroid cancer	4660.1	177.6	10.2	39.8	0	0	0	0	4660.1	177.6	10.2	39.8
**Neoplasms**	**462545.0**	**160.2**	**4.6**	**48.5**	**419338.0**	**179.4**	**4.2**	**59.6**	**865438.8**	**167.5**	**8.6**	**52.7**

Population attributable fraction (PAF): Percentages of death cases attributable to risk factors.

In 2019, cancer deaths attributable to a high body mass index were mainly gastrointestinal cancers such as colon and rectum cancer, esophageal cancer and pancreatic cancer. Cancer deaths attributable to a high fasting plasma glucose were mainly respiratory system cancers and digestive system cancers such as tracheal, bronchus, and lung cancer, colon and rectum cancer, and pancreatic cancer. In addition, we found that Africa and Asia other than South Asia accounted for the vast majority of gastrointestinal cancer deaths attributable to a high body mass index. East Asia and high-income North America had the highest mortality burden of respiratory system cancers attributable to a high fasting plasma glucose (**Figures 5B, C**).

## Discussion

Metabolic factors are important risk factors for cancer mortality and are also a focus of cancer prevention and control. In the past 30 years, under global cancer prevention and control policies, the cancer burden attributable to most factors, such as tobacco, alcohol use, dietary risks, and environmental risks, has shown a downward trend to varying degrees. However, the cancer burden attributable to metabolic factors shows a clear upward trend. Our study is based on the latest data from GBD2019; to our knowledge, it is the only study that has conducted in-depth and detailed research on the cancer burden attributable to metabolic factors.

In 2019, the number of cancer deaths attributable to metabolic factors was 0.87 million, an increase of 167.45% over 1990, and the ASDR was 10.74 per 100,000, an increase of 23.88% over 1990. The increase in the number of deaths was significantly higher than the increase in the ASDR, indicating that population growth and aging also contributed to the increase in the absolute value. The cancer burden attributable to metabolic factors has become an important public health issue worldwide. Due to the lack of control of high fasting plasma glucose and high body mass index in low-SDI regions, the gap in the related cancer mortality burden between low-SDI regions and high-SDI regions has gradually narrowed.

In high SDI regions such as Australia and the United States, the incidence of metabolic diseases has also risen sharply with economic development. Nevertheless, due to the successful transition of the economy and the government’s gradual emphasis on basic health, the mortality rate related to metabolic diseases has decreased in recent years (14, 15). For example, as early as 2000, the United States began screening young people aged 10 years and above for fasting plasma glucose levels, and by 2018, the screening targeted high-risk youth with a high body mass index and ≥2 risk factors, and found a quarter of the U.S. youth were eligible for prediabetes screening (16). Australia’s National Health and Medical Research Council developed clinical practice guidelines for managing overweight and obesity in Australian adults, adolescents and children (17). Under the abovementioned health policies, the incidence of diabetes (18) and obesity (19) in many high-income regions has remained stable, so the increase in cancer burden attributable to metabolic factors is not obvious. In contrast, in lower SDI regions, such as the Middle East and North Africa, the Gulf Cooperation Council greatly promoted the prosperity of the energy and oil industries (20), and rapid economic transformation, industrialization, urbanization and globalization have brought about dramatic changes, reduced opportunities for physical activity, and unusual changes in dietary patterns (21) toward a “Western diet” characterized by high intakes of refined carbohydrates, added sugars, fats and animal-sourced foods (22), leading to a significant increase in the prevalence of diabetes (23) and a high body-mass index (19), with a corresponding increase in the cancer burden. At the same time, suboptimal health care services (24), limited access to large-scale cancer screening programs (25) and poverty (26) also contribute to the rising cancer burden. Therefore, low SDI regions need to pay more attention to the cancer burden attributable to metabolic factors. The government needs to equip the health care system to support the primary prevention of metabolic diseases and strengthen education about healthy lifestyles.

Significant heterogeneity in the burden by sex in different regions was observed. In lower SDI regions, the burden on women is significantly higher than on men, which is diametrically the opposite of higher SDI regions. Occupation is a significant source of physical activity in many lower SDI regions. In South Asia, North Africa, the Middle East, Sub-Saharan Africa, Latin America and the Caribbean, men have significantly higher daily physical activity levels than women (27–32). The influx of refined carbohydrates and sweet foods, along with sex differences in related metabolic capabilities, contributes to a greater increase in triglyceride levels (33) and fasting plasma glucose levels in women. Therefore, in lower SDI regions, women are more likely to have a higher body mass index (34), leading to a corresponding increase in their cancer mortality burden. Increasing awareness of metabolic factors, better obesity management and fasting plasma glucose level monitoring are essential to narrow the sex gap.

In high SDI regions, the main death burden is concentrated in the age group of 70 years and above, which is closely related to the significant aging trend and the improvement of medical care standards in high SDI regions (35). In contrast, in low-SDI regions, the death burden population is mainly concentrated among people aged 50-69 years, and the trend of premature death is significantly higher than that in high-SDI regions. Poor health infrastructure (36), ineffective screening or lack of early application of effective screening methods and poor access to effective treatment (37) are probably responsible for this difference. Younger populations are the most economically productive members of society, and improving cancer prevention and diagnostic efficacy would not only lead to a huge societal impact but could also be cost-effective (38). Therefore, in low SDI regions, the government urgently needs to strengthen the construction of health infrastructure, encourage young people to engage in outdoor sports, reverse poor dietary patterns, and carry out management and screening of young groups for a high fasting plasma glucose and a high body mass index.

Our results show that the common main cancer type of cancer mortality burden attributable to metabolic factors is digestive system cancer (accounting for 50.11%), which is the core burden. Many epidemiological studies have consistently found that obese individuals have an increased risk of esophageal adenocarcinoma and colorectal cancer (39). Compared with nonhyperglycemic patients, gastrointestinal patients with elevated fasting plasma glucose have a higher risk of death (40), which may be related to increased circulating insulin levels (41) and chronic inflammation (42). In addition, high fasting plasma glucose and high body mass index are also closely related to the incidence of lung cancer and breast cancer (43–45). Therefore, controlling and preventing high fasting plasma glucose and high body mass index is an urgent problem to be solved in cancer prevention and control.

## Conclusion

In conclusion, the global burden of cancer mortality attributable to metabolic factors has increased dramatically. Higher SDI regions have a higher burden, but the burden is shifting to lower SDI regions due to the increasing trend in lower SDI regions. Significant regional heterogeneity in sex differences was observed, and the burden on women in low SDI regions was significant. Metabolic-related cancer mortality tends to occur at younger ages, with a heavy premature death burden in low SDI regions. Gastrointestinal cancers are at the core of the current cancer mortality burden attributable to metabolic factors. Policies should be adopted in the future to reduce the burden of high body mass index and high fasting plasma glucose in the general population.

## Data availability statement

Publicly available datasets were analyzed in this study. This data can be found here: http://ghdx.healthdata.org/gbd-results-tool.

## Author contributionss

YZ contributed to study design, data collection, data analysis, data interpretation, and writing of the manuscript. YD contributed study conduct, data collection, and writing of the manuscript. NZ contributed data collection, analysis, and interpretation. MM contributed review, interpretation, and analysis. YL contributed data collection, data analysis, and data interpretation. JZ contributed data collection, data analysis, and data interpretation. SW and YY contributed study concept and design, chairing of steering committee, oversight of study implementation, extensive data analysis and interpretation, and writing and approval of final version of manuscript. All authors contributed to the article and approved the submitted version.
