# Supplementary figures and images for: Emerging patterns and trends in global cancer burden attributable to metabolic factors, based on the Global Burden of Disease Study 2019
Source: Front Oncol. 2023 Jan 19;13:1032749. doi: 10.3389/fonc.2023.1032749 (PMC9893408; doi:10.3389/fonc.2023.1032749)

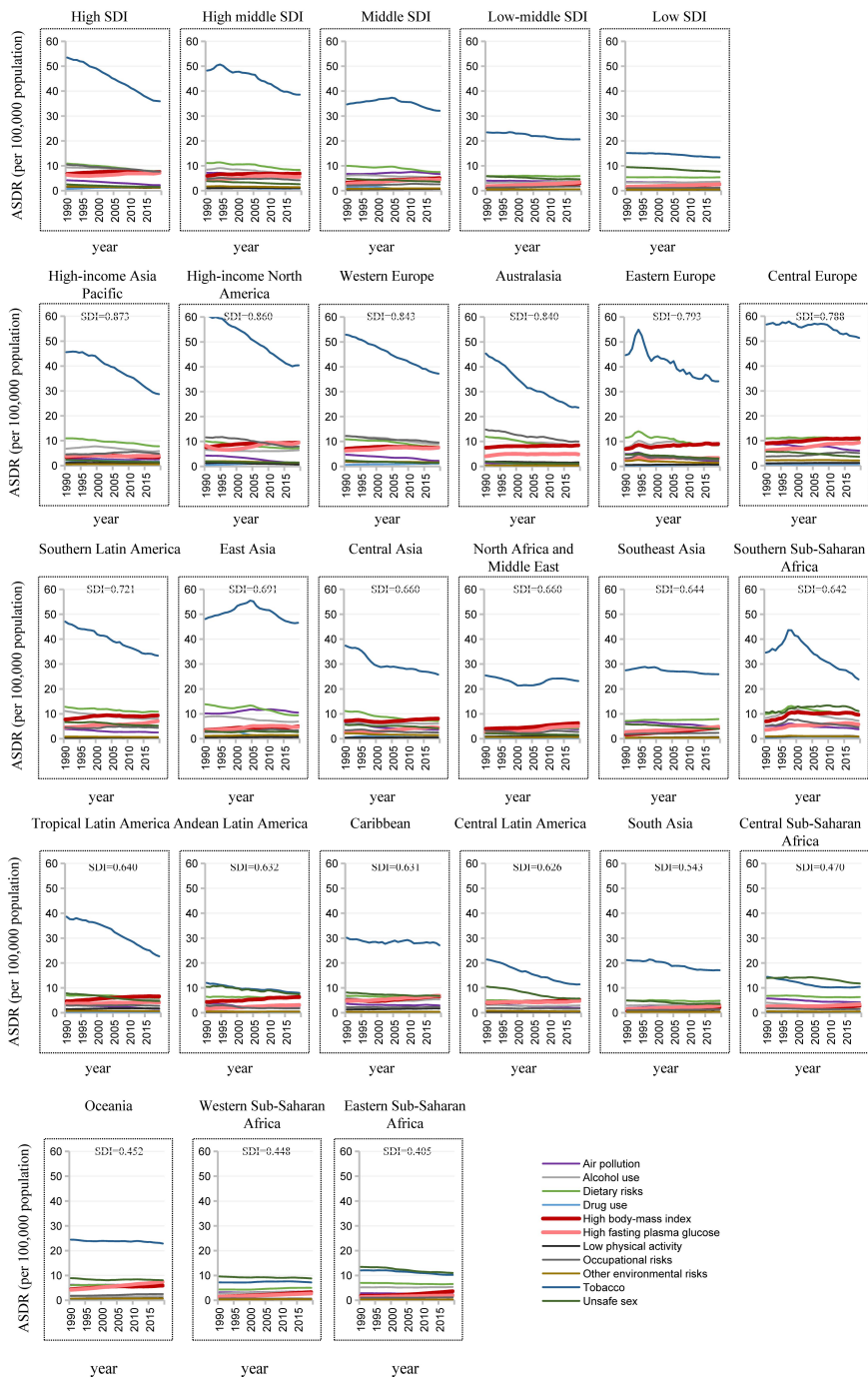

Supplement: Supplementary file 1 [file DataSheet_1.pdf]

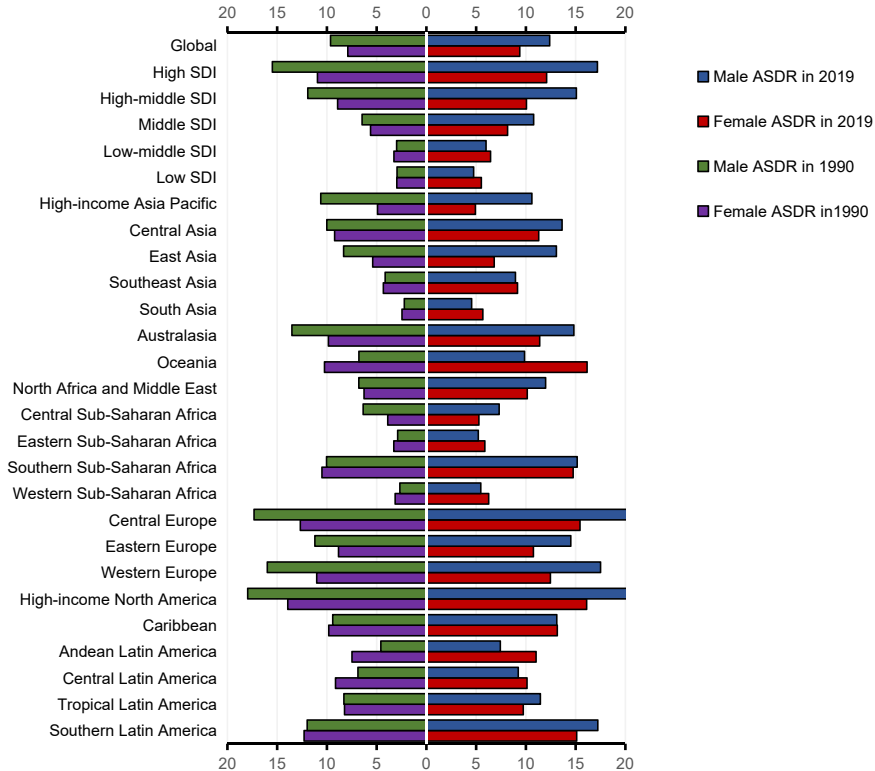

Supplement: Supplementary file 2 [file DataSheet_2.pdf]

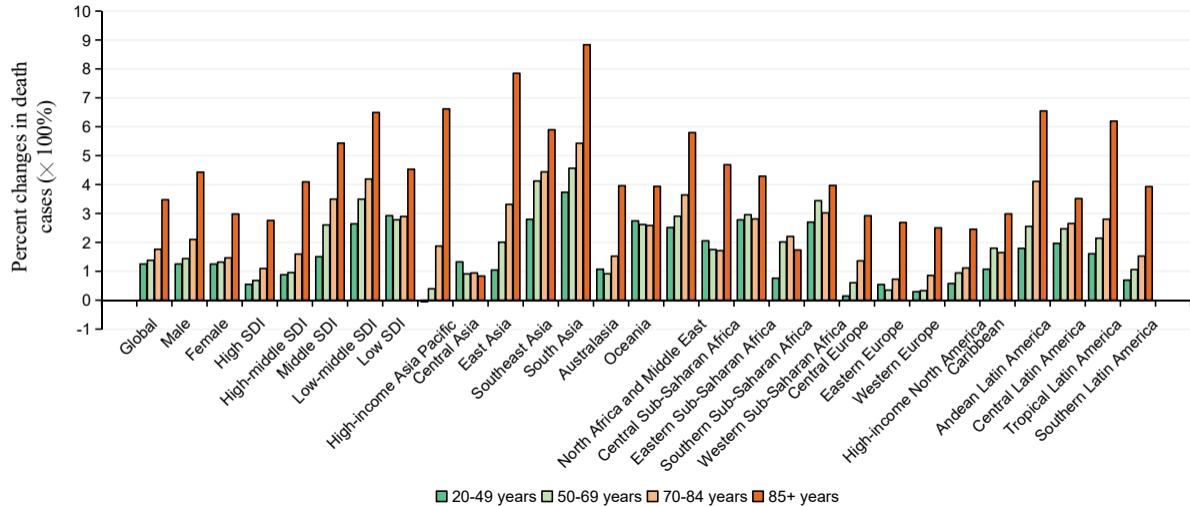

Supplement: Supplementary file 3 [file DataSheet_3.pdf]

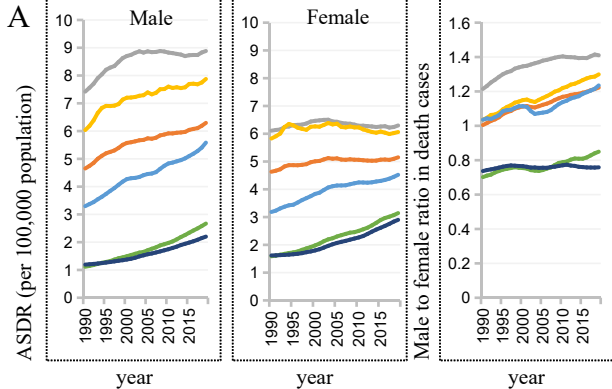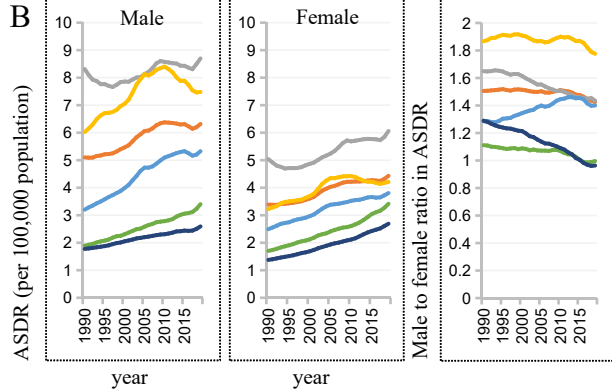

Global High SDI High-middle SDI Middle SDI Low-middle SDI Low SDI

Supplement: Supplementary file 4 [file DataSheet_4.pdf]
