# Supplementary material for: Emerging patterns and trends in global cancer burden attributable to metabolic factors, based on the Global Burden of Disease Study 2019
Source: Front Oncol. 2023 Jan 19;13:1032749. doi: 10.3389/fonc.2023.1032749 (PMC9893408; doi:10.3389/fonc.2023.1032749)
Supplement: Supplementary file 5 [file DataSheet_5.pdf]

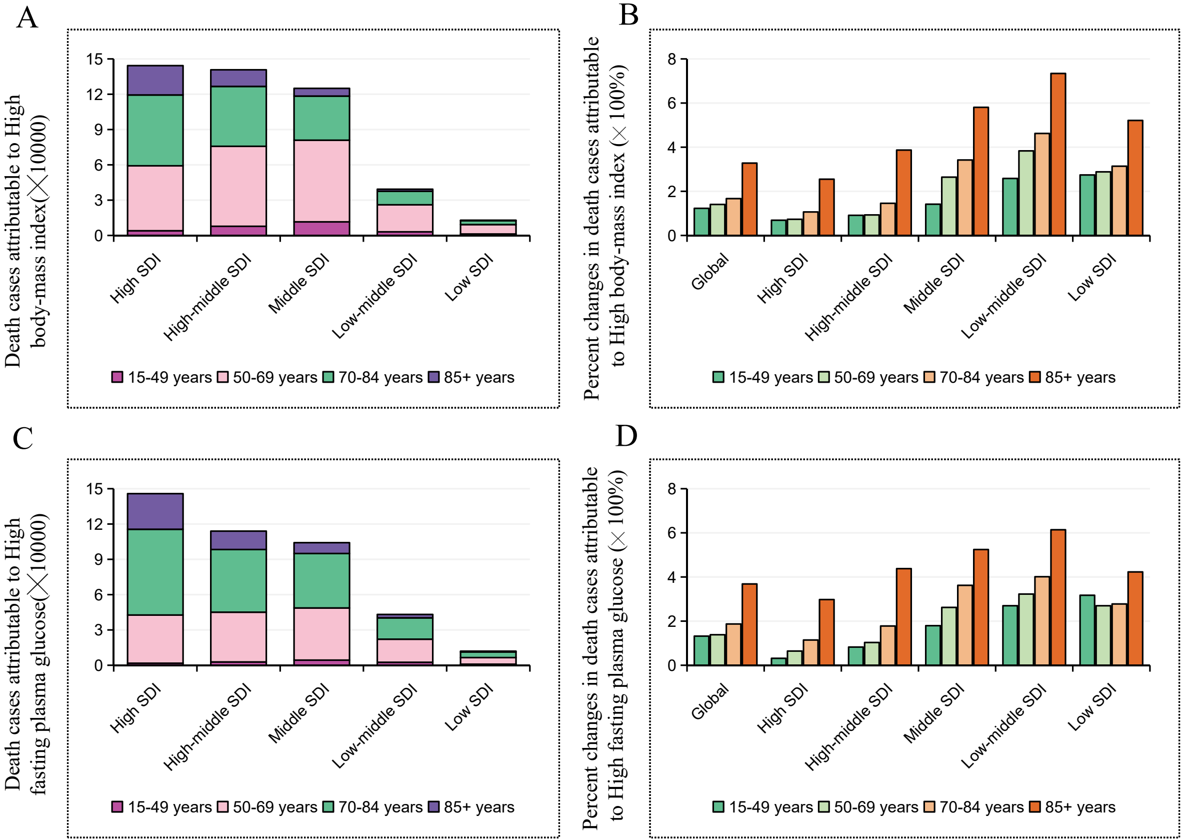
A
B
C
D
Death cases attributable to High body-mass index(×10000)
Percent changes in death cases attributable to High body-mass index (× 100%)
Death cases attributable to High fasting plasma glucose(×10000)
Percent changes in death cases attributable to High fasting plasma glucose (× 100%)
High SDI
High-middle SDI
Middle SDI
Low-middle SDI
Low SDI
Global
15-49 years
50-69 years
70-84 years
85+ years
