# Supplementary material for: Emerging patterns and trends in global cancer burden attributable to metabolic factors, based on the Global Burden of Disease Study 2019
Source: Front Oncol. 2023 Jan 19;13:1032749. doi: 10.3389/fonc.2023.1032749 (PMC9893408; doi:10.3389/fonc.2023.1032749)
Supplement: Supplementary file 6 [file DataSheet_6.zip › Raw data/Statistical programs.docx]

| Title | Statistical programs |
| --- | --- |
| Figure S1 | Excel |
| Figure S2 | Excel |
| Figure S3 | Excel |
| Figure S4 | Excel |
| Figure S5 | Excel |
| Figure 1-A | Excel |
| Figure 1-B | Excel |
| Figure 1-C | Excel |
| Figure 1-D | R language software |
| Figure 2-A | Excel |
| Figure 2-B | Excel |
| Figure 2-C | Excel |
| Figure 2-D | R language software |
| Figure 2-E | R language software, Joinpoint 4.2.0.1 |
| Figure 2-F | R language software |
| Figure 3-A | Excel |
| Figure 3-B | Excel |
| Figure 3-C | Excel |
| Figure 3-D | Excel |
| Figure 4-A | Excel |
| Figure 4-B | Excel |
| Figure 4-C | Excel |
| Figure 5-A | Excel |
| Figure 5-B, Figure 5-C | Excel |
| Table 1 | Excel, Joinpoint 4.2.0.1 |
| Table 2 | Excel |
| Table S1 | Excel, Joinpoint 4.2.0.1 |
