# Supplementary material for: Emerging patterns and trends in global cancer burden attributable to metabolic factors, based on the Global Burden of Disease Study 2019
Source: Front Oncol. 2023 Jan 19;13:1032749. doi: 10.3389/fonc.2023.1032749 (PMC9893408; doi:10.3389/fonc.2023.1032749)
Supplement: Supplementary file 7 [file Table_1.docx]

**Table S1. Age-standardized SEV rate of metabolic factors by GBD regions in 1990 and 2019, and their variations from 1990 to 2019**

|  |  | **High body-mass index** | | |  | **High fasting plasma glucose** | | |
| --- | --- | --- | --- | --- | --- | --- | --- | --- |
| **Location** | **Sex** | **1990 (95% UI)** | **2019 (95% UI)** | **AAPC (95%CI)** |  | **1990 (95% UI)** | **2019 (95% UI)** | **AAPC (95%CI)** |
| Global | Both | 11.09(7.96-15.23) | 19.45(15.57-24.39) | 1.99(1.98-1.99) |  | 7.88(6.96-8.85) | 11.72(10.56-12.94) | 1.46(1.44-1.49) |
|  | Male | 9.67(6.59-13.71) | 18.26(14.22-23.38) | 2.25(2.24-2.25) |  | 8.33(7.33-9.38) | 12.7(11.44-14.02) | 1.54(1.52-1.56) |
|  | Female | 12.44(9.05-17.05) | 20.59(16.66-25.75) | 1.78(1.78-1.79) |  | 7.46(6.64-8.35) | 10.81(9.74-11.96) | 1.38(1.36-1.4) |
| **Socio-demographic index** |  |  |  |  |  |  |  |  |
| High SDI | Both | 19.27(14.41-25.31) | 30.94(24.88-37.92) | 1.63(1.59-1.67) |  | 8.43(7.49-9.36) | 12.8(11.66-13.99) | 1.75(1.71-1.8) |
|  | Male | 18.1(12.99-24.71) | 30.21(23.08-38.14) | 1.78(1.73-1.82) |  | 9.44(8.34-10.55) | 14.45(13.16-15.78) | 1.76(1.72-1.8) |
|  | Female | 20.31(15.43-26.58) | 31.66(25.84-38.29) | 1.51(1.47-1.55) |  | 7.53(6.7-8.37) | 11.24(10.22-12.37) | 1.72(1.66-1.78) |
| High-middle SDI | Both | 14.05(10.35-18.86) | 23.2(18.43-29.42) | 1.81(1.79-1.82) |  | 7.76(6.84-8.72) | 10.75(9.62-11.9) | 1.33(1.28-1.38) |
|  | Male | 12.31(8.71-17.04) | 22.8(17.79-29.21) | 2.23(2.22-2.25) |  | 8.22(7.22-9.25) | 11.63(10.38-12.89) | 1.39(1.34-1.44) |
|  | Female | 15.57(11.5-20.99) | 23.42(18.6-29.76) | 1.46(1.45-1.48) |  | 7.35(6.48-8.24) | 9.95(8.88-11.03) | 1.26(1.21-1.3) |
| Middle SDI | Both | 9.01(6.27-12.76) | 20.07(16.21-24.94) | 2.86(2.85-2.87) |  | 7.98(7.04-8.98) | 11.57(10.41-12.78) | 1.27(1.25-1.29) |
|  | Male | 7.72(5.07-11.4) | 18.76(14.76-23.8) | 3.17(3.16-3.17) |  | 8.07(7.1-9.13) | 12.21(10.94-13.53) | 1.43(1.41-1.45) |
|  | Female | 10.33(7.28-14.48) | 21.34(17.37-26.65) | 2.6(2.58-2.62) |  | 7.9(6.97-8.88) | 10.98(9.84-12.15) | 1.12(1.09-1.14) |
| Low-middle SDI | Both | 5.51(3.62-8.33) | 13.86(11-17.39) | 3.33(3.32-3.35) |  | 7.51(6.59-8.54) | 12.75(11.29-14.25) | 1.68(1.66-1.7) |
|  | Male | 4.7(2.89-7.32) | 12.58(9.73-16.14) | 3.55(3.52-3.58) |  | 7.87(6.9-9) | 13.81(12.19-15.46) | 1.79(1.76-1.81) |
|  | Female | 6.36(4.19-9.4) | 15.12(12.04-19.07) | 3.14(3.13-3.15) |  | 7.14(6.27-8.09) | 11.74(10.35-13.16) | 1.58(1.56-1.6) |
| Low SDI | Both | 5.76(3.74-8.56) | 11.37(8.71-14.68) | 2.47(2.43-2.52) |  | 6.62(5.77-7.52) | 10.48(9.12-11.9) | 1.45(1.43-1.46) |
|  | Male | 4.79(2.95-7.47) | 9.54(6.92-12.72) | 2.46(2.42-2.51) |  | 7.08(6.17-8.11) | 11.24(9.74-12.89) | 1.41(1.39-1.43) |
|  | Female | 6.75(4.4-9.75) | 13.18(10.14-16.93) | 2.46(2.43-2.5) |  | 6.16(5.38-6.99) | 9.75(8.52-11.04) | 1.5(1.49-1.51) |
| **GBD Regions** |  |  |  |  |  |  |  |  |
| High-income Asia Pacific | Both | 9.03(5.69-13.64) | 13(9.26-17.8) | 1.14(1.07-1.2) |  | 6.88(6.02-7.8) | 8.41(7.39-9.56) | 0.39(0.35-0.44) |
|  | Male | 8.96(5.51-13.66) | 14.01(9.87-19.34) | 1.43(1.34-1.51) |  | 8.43(7.39-9.59) | 10.25(8.99-11.67) | 0.44(0.4-0.49) |
|  | Female | 9.04(5.34-13.92) | 11.89(7.97-16.86) | 0.81(0.77-0.85) |  | 5.57(4.86-6.32) | 6.68(5.88-7.58) | 0.23(0.18-0.28) |
| Central Asia | Both | 19.31(14.7-25.34) | 29.43(23.69-37.24) | 1.57(1.5-1.64) |  | 5.88(5.2-6.64) | 12.68(11.34-14.08) | 3.03(2.99-3.07) |
|  | Male | 16.23(11.77-21.85) | 26.88(20.91-34.56) | 1.87(1.79-1.95) |  | 5.62(4.92-6.37) | 12.72(11.23-14.22) | 3.17(3.14-3.21) |
|  | Female | 21.86(16.65-29.27) | 31.59(25.12-40) | 1.38(1.31-1.44) |  | 6.02(5.33-6.83) | 12.62(11.29-14.05) | 2.97(2.92-3.01) |
| East Asia | Both | 4.81(2.41-8.52) | 13.15(9.15-18.36) | 3.6(3.59-3.61) |  | 7.6(6.6-8.68) | 9.45(8.33-10.57) | 0.92(0.85-0.98) |
|  | Male | 4.61(2.26-8.19) | 14.04(9.78-19.37) | 4.01(3.99-4.02) |  | 7.64(6.59-8.73) | 10.15(8.97-11.44) | 1.16(1.09-1.22) |
|  | Female | 5.01(2.47-8.84) | 12.14(8-17.65) | 3.15(3.14-3.16) |  | 7.54(6.54-8.69) | 8.76(7.7-9.85) | 0.65(0.59-0.72) |
| Southeast Asia | Both | 5.07(3.11-7.76) | 14.49(11.5-17.85) | 3.86(3.84-3.89) |  | 6.4(5.64-7.22) | 10.42(9.32-11.59) | 1.49(1.45-1.54) |
|  | Male | 4.38(2.58-6.89) | 12.84(9.93-16.01) | 3.79(3.78-3.8) |  | 6.46(5.65-7.3) | 10.56(9.36-11.83) | 1.53(1.48-1.57) |
|  | Female | 5.74(3.57-8.54) | 16.08(12.85-19.83) | 3.92(3.88-3.96) |  | 6.37(5.62-7.21) | 10.31(9.23-11.46) | 1.47(1.43-1.51) |
| South Asia | Both | 4.23(2.69-6.41) | 11.45(8.79-13.99) | 3.56(3.53-3.59) |  | 8.1(7.07-9.27) | 14.22(12.53-16.05) | 1.72(1.69-1.75) |
|  | Male | 3.72(2.28-5.8) | 10.79(8.14-13.62) | 3.8(3.77-3.83) |  | 8.6(7.53-9.86) | 15.53(13.64-17.59) | 1.79(1.76-1.82) |
|  | Female | 4.79(2.97-7.07) | 12.1(9.41-14.92) | 3.32(3.28-3.37) |  | 7.54(6.57-8.59) | 12.91(11.27-14.63) | 1.67(1.64-1.69) |
| Australasia | Both | 23.02(17.33-30.86) | 35.69(28.32-44.06) | 1.48(1.44-1.52) |  | 4.56(4.06-5.12) | 8.15(7.06-9.31) | 1.89(1.82-1.95) |
|  | Male | 22.06(16.13-29.66) | 33.5(25.33-43.52) | 1.47(1.44-1.49) |  | 5.07(4.49-5.78) | 9.32(7.9-10.88) | 2.05(2-2.1) |
|  | Female | 23.91(17.61-32.63) | 37.78(30.58-45.23) | 1.5(1.44-1.55) |  | 4.15(3.64-4.7) | 7.08(6.16-8.2) | 1.67(1.6-1.75) |
| Oceania | Both | 14.28(9.76-19.9) | 17.26(12.66-23.08) | 0.37(0.29-0.45) |  | 13.73(12.22-15.55) | 23.95(21.63-26.43) | 1.98(1.95-2.01) |
|  | Male | 11.65(7.35-17.16) | 14.8(10.29-20.49) | 0.56(0.48-0.63) |  | 15.32(13.44-17.63) | 25.92(23.06-28.73) | 1.84(1.81-1.87) |
|  | Female | 17.11(12.14-23.49) | 19.89(14.75-26.25) | 0.23(0.15-0.31) |  | 12.11(10.75-13.77) | 21.88(19.65-24.49) | 2.13(2.1-2.16) |
| North Africa and Middle East | Both | 18.93(14.29-24.89) | 33.31(26.43-41.45) | 2(2-2.01) |  | 7.91(6.97-8.92) | 15.22(13.66-16.79) | 2.6(2.56-2.65) |
|  | Male | 15.25(11-20.61) | 30.7(23.62-39.12) | 2.5(2.49-2.51) |  | 7.92(6.94-8.92) | 15.48(13.83-17.26) | 2.64(2.6-2.69) |
|  | Female | 22.78(17.41-29.86) | 36.1(28.61-44.48) | 1.62(1.61-1.62) |  | 7.89(7-8.86) | 14.93(13.45-16.46) | 2.57(2.53-2.61) |
| Central Sub-Saharan Africa | Both | 7.75(5.33-10.67) | 11.66(8.92-14.94) | 1.1(1-1.21) |  | 7.97(6.9-9.15) | 11.17(9.66-12.68) | 1.21(1.2-1.22) |
|  | Male | 6.95(4.6-9.86) | 10.38(7.57-13.72) | 0.99(0.86-1.11) |  | 9.47(8.08-10.96) | 13.74(11.87-15.77) | 1.34(1.31-1.36) |
|  | Female | 8.53(5.75-11.78) | 12.94(9.7-16.7) | 1.21(1.11-1.3) |  | 6.62(5.72-7.65) | 9.09(7.79-10.42) | 1.13(1.13-1.14) |
| Eastern Sub-Saharan Africa | Both | 7.37(5.02-10.54) | 14.39(11.42-18.04) | 2.48(2.41-2.55) |  | 5.18(4.53-5.88) | 6.77(5.94-7.68) | 0.93(0.92-0.95) |
|  | Male | 6.03(3.85-9.04) | 12.06(9.13-15.58) | 2.53(2.46-2.6) |  | 5.59(4.89-6.35) | 7.66(6.67-8.71) | 1.11(1.09-1.13) |
|  | Female | 8.7(5.86-12.26) | 16.63(13.19-20.91) | 2.44(2.37-2.5) |  | 4.81(4.22-5.47) | 6(5.29-6.83) | 0.76(0.75-0.77) |
| Southern Sub-Saharan Africa | Both | 20.92(16.54-26.09) | 31.34(25.67-37.64) | 1.46(1.45-1.47) |  | 8.11(7.09-9.23) | 13.08(11.67-14.42) | 1.77(1.73-1.82) |
|  | Male | 13.15(9.76-17.48) | 21.67(17.38-27.3) | 1.79(1.77-1.81) |  | 7.69(6.65-8.82) | 12.28(10.91-13.69) | 1.78(1.73-1.82) |
|  | Female | 27.79(21.72-34.96) | 40.09(31.82-47.87) | 1.33(1.33-1.34) |  | 8.48(7.44-9.62) | 13.76(12.3-15.15) | 1.76(1.7-1.82) |
| Western Sub-Saharan Africa | Both | 8.09(5.51-11.51) | 18.55(14.94-23.36) | 2.86(2.83-2.88) |  | 4.87(4.22-5.54) | 7.5(6.57-8.5) | 1.49(1.48-1.49) |
|  | Male | 6.93(4.36-10.08) | 15.87(12.25-20.54) | 2.84(2.83-2.86) |  | 4.89(4.24-5.58) | 7.53(6.57-8.51) | 1.5(1.49-1.51) |
|  | Female | 9.32(6.52-12.98) | 21.01(17.05-26.16) | 2.79(2.75-2.83) |  | 4.89(4.26-5.57) | 7.48(6.54-8.51) | 1.43(1.41-1.44) |
| Central Europe | Both | 20.77(15.74-27.47) | 30.15(24.02-37.99) | 1.33(1.33-1.34) |  | 8.15(7.19-9.16) | 12.72(11.42-14.05) | 1.64(1.61-1.67) |
|  | Male | 20.19(14.89-27.02) | 30.8(23.61-39.86) | 1.52(1.51-1.53) |  | 8.79(7.74-10) | 14.55(12.99-16.15) | 1.83(1.8-1.87) |
|  | Female | 21.12(16.05-27.99) | 29.32(23.73-36.81) | 1.17(1.17-1.18) |  | 7.55(6.66-8.49) | 11.07(9.96-12.29) | 1.44(1.41-1.46) |
| Eastern Europe | Both | 20.9(16.58-26.54) | 29.27(23.84-36.17) | 1.29(1.24-1.33) |  | 6.56(5.59-7.62) | 8.16(7.06-9.31) | 0.75(0.72-0.78) |
|  | Male | 16.92(12.92-21.59) | 26.61(21.03-33.86) | 1.75(1.69-1.8) |  | 7.34(6.13-8.63) | 8.78(7.57-10.21) | 0.56(0.53-0.59) |
|  | Female | 23.93(18.89-30.69) | 31.18(25.27-39.09) | 0.99(0.95-1.02) |  | 5.92(5.04-6.9) | 7.62(6.54-8.68) | 0.9(0.88-0.93) |
| Western Europe | Both | 18.35(13.85-24.06) | 26.46(21.05-33.75) | 1.24(1.21-1.28) |  | 7.9(7.01-8.87) | 12.69(11.41-14.04) | 1.6(1.59-1.61) |
|  | Male | 18.17(13.26-24.14) | 25.47(19.11-33.2) | 1.12(1.08-1.16) |  | 8.43(7.41-9.56) | 14.18(12.72-15.71) | 1.72(1.69-1.74) |
|  | Female | 18.4(13.83-24.63) | 27.41(22.43-34.5) | 1.38(1.35-1.42) |  | 7.41(6.57-8.32) | 11.28(10.1-12.56) | 1.46(1.44-1.48) |
| High-income North America | Both | 26.81(20.11-34.83) | 41.05(32-49.52) | 1.45(1.39-1.51) |  | 10.5(9.34-11.73) | 15.67(14.44-16.97) | 2.32(2.2-2.44) |
|  | Male | 24(17.02-32.76) | 39.08(29.45-49.08) | 1.7(1.63~1.77) |  | 11.84(10.43-13.23) | 17.49(16.1-18.97) | 2.21(2.1-2.32) |
|  | Female | 29.38(22.06-37.25) | 42.95(34.01-50.72) | 1.26(1.2~1.32) |  | 9.34(8.3-10.37) | 14.03(12.87-15.28) | 2.43(2.29-2.56) |
| Caribbean | Both | 18.33(14.19-23.74) | 26.17(20.51-33) | 1.4(1.37-1.42) |  | 11.24(10.15-12.42) | 15.88(14.47-17.38) | 1.17(1.16-1.18) |
|  | Male | 15.28(11.34-20.27) | 22.83(17.21-29.81) | 1.56(1.53-1.58) |  | 11.45(10.17-12.72) | 16.44(14.89-18.05) | 1.23(1.22-1.24) |
|  | Female | 21.27(16.44-27.76) | 29.4(23.02-36.7) | 1.29(1.26-1.31) |  | 11.06(9.93-12.27) | 15.39(14.03-16.85) | 1.11(1.1-1.12) |
| Andean Latin America | Both | 19.52(14.44-25.91) | 32.07(24.33-41) | 1.7(1.69-1.71) |  | 5.53(4.84-6.22) | 9.43(8.31-10.62) | 1.85(1.83-1.87) |
|  | Male | 16.93(11.9-23.6) | 28.76(21.36-37.9) | 1.79(1.78-1.81) |  | 5.46(4.76-6.18) | 9.57(8.38-10.88) | 1.96(1.94-1.97) |
|  | Female | 22.07(16.28-29.43) | 35.33(26.67-44.92) | 1.63(1.62-1.63) |  | 5.6(4.93-6.33) | 9.3(8.18-10.45) | 1.75(1.73-1.78) |
| Central Latin America | Both | 20.26(14.76-27.15) | 30.57(23.5-39.48) | 1.42(1.39-1.45) |  | 13.99(12.69-15.25) | 18.4(16.81-19.95) | 1(0.98-1.01) |
|  | Male | 18.49(13.1-25.12) | 28.57(21.5-37.26) | 1.54(1.51-1.57) |  | 14.07(12.64-15.43) | 19.74(17.97-21.35) | 1.33(1.31-1.36) |
|  | Female | 21.93(15.92-29.91) | 32.38(24.63-41.89) | 1.32(1.29-1.35) |  | 13.9(12.63-15.14) | 17.22(15.71-18.71) | 0.68(0.66-0.7) |
| Tropical Latin America | Both | 14.9(10.46-20.59) | 31.16(25.45-39.39) | 2.8(2.76-2.84) |  | 9.84(8.66-11.04) | 11.42(10.12-12.79) | 0.8(0.75-0.85) |
|  | Male | 13.45(9.11-19.57) | 30.33(23.93-38.78) | 3.11(3.06-3.15) |  | 10.22(8.93-11.53) | 12.28(10.8-13.9) | 0.99(0.93-1.05) |
|  | Female | 16.27(11.41-22.77) | 31.85(26.07-39.86) | 2.53(2.5-2.56) |  | 9.53(8.33-10.76) | 10.71(9.46-12) | 0.63(0.59-0.68) |
| Southern Latin America | Both | 14.29(9.68-20.31) | 26.17(19.68-34.74) | 2.04(1.97-2.12) |  | 6.53(5.74-7.39) | 11.64(10.21-13.08) | 1.91(1.89-1.94) |
|  | Male | 13.37(8.55-19.37) | 24.52(17.64-32.78) | 2.08(2.01-2.14) |  | 6.48(5.66-7.38) | 12.64(10.83-14.42) | 2.16(2.13-2.2) |
|  | Female | 15.1(10.2-21.87) | 27.65(20.76-36.81) | 2.02(1.94-2.1) |  | 6.53(5.66-7.5) | 10.81(9.51-12.23) | 1.72(1.69-1.74) |
| AAPC: average annual percentage change, GBD: Global Disease Burden,SDI: Socio-demographic Index, UI: uncertainty interval | | | | | | | | |
